# Isolation and purification of all-*trans* diadinoxanthin and all-*trans* diatoxanthin from diatom *Phaeodactylum tricornutum*

**DOI:** 10.1007/s10811-016-0961-x

**Published:** 2016-09-27

**Authors:** Paulina Kuczynska, Malgorzata Jemiola-Rzeminska

**Affiliations:** 1 Department of Plant Physiology and Biochemistry, Faculty of Biochemistry, Biophysics and Biotechnology, Jagiellonian University, Gronostajowa 7, 30-387 Krakow, Poland; 2 Malopolska Centre of Biotechnology, Gronostajowa 7A, 30-387, Krakow, Poland

**Keywords:** Carotenoids, Chromatography, Diadinoxanthin, Diatoms, Diatoxanthin, *Phaeodactylum tricornutum*

## Abstract

Two diatom-specific carotenoids are engaged in the diadinoxanthin cycle, an important mechanism which protects these organisms against photoinhibition caused by absorption of excessive light energy. A high-performance and economical procedure of isolation and purification of diadinoxanthin and diatoxanthin from the marine diatom *Phaeodactylum tricornutum* using a four-step procedure has been developed. It is based on the use of commonly available materials and does not require advanced technology. Extraction of pigments, saponification, separation by partition and then open column chromatography, which comprise the complete experimental procedure, can be performed within 2 days. This method allows HPLC grade diadinoxanthin and diatoxanthin of a purity of 99 % or more to be obtained, and the efficiency was estimated to be 63 % for diadinoxanthin and 73 % for diatoxanthin. Carefully selected diatom culture conditions as well as analytical ones ensure highly reproducible performance. A protocol can be used to isolate and purify the diadinoxanthin cycle pigments both on analytical and preparative scale.

**Electronic supplementary material:**

The online version of this article (doi:10.1007/s10811-016-0961-x) contains supplementary material, which is available to authorized users.

## Introduction

Carotenoids are the most widespread group of naturally occurring pigments composed of more than 700 structurally different compounds (Britton et al. 2004). They typically consist of C_40_ hydrocarbon backbone and are divided into two classes, carotenes—polyunsaturated hydrocarbons and xanthophylls—their oxygenated derivatives. With conjugated double-bond system, carotenoids can absorb light in the visible range of the spectrum. This also causes the compounds to be deeply coloured; most of the carotenes are reddish and most of the xanthophylls are yellow-orange.

In photosynthetic organisms, carotenoids have two well-recognized functions: one, as accessory light-harvesting pigments and, the other, as quenchers of singlet oxygen and chlorophyll triplet states to provide protection against photooxidative damage (Vílchez et al. 2011). Lycopene and β,β-carotene (ββ-Car) are found in light-harvesting complexes (LHC) in plants and green algae (Koepke et al. 1996; Scheer 2003), while fucoxanthin (Fuco) is abundantly present in fucoxanthin-chlorophyll protein (FCP) complexes in diatoms (for review see Kuczynska et al. (2015)). The photoprotective role of carotenoids is based on their ability to quench excited molecules and scavenge free radicals to prevent the damage of photosynthetic apparatus caused by excess light energy. Some xanthophylls are capable of cyclic conversions through de-epoxidation and epoxidation, referred to as the xanthophyll cycle. Violaxanthin (Viola), antheraxanthin (Anth) and zeaxanthin (Zea) are engaged in the most common violaxanthin cycle (VAZ cycle) which occurs in plants, mosses, lichens and algae (Yamamoto et al. 1962). In the primitive green alga *Mantoniella squamata*, the specific type of VAZ cycle has been described by Goss et al. (1998) and Gilmore and Yamamoto (2001), with Anth as the main product of de-epoxidation instead of Zea. On the other hand, diatoms display the diadinoxanthin cycle (DD cycle) in which interconversion between epoxidised diadinoxanthin (Diadino) and epoxy-free diatoxanthin (Diato) occurs. However, the VAZ cycle can be also observed under specific conditions (Lohr & Wilhelm 1999). Finally, in other organisms, the lutein epoxide cycle engaging lutein and lutein epoxide (Rabinowitch et al. 1975), as well as the siphonaxanthin cycle with lutein and siphonaxanthin (Raniello et al. 2006), were identified.

Carotenoids are natural bioactive compounds. Due to their beneficial activities such as antioxidant, anticancer, anti-inflammatory, anti-obesity and neuroprotective effects, they are of great importance for the food, cosmetic and pharmaceutical industries (Pangestuti & Kim 2011). Carotenoid production both by extraction from plants and subsequent purification or through chemical synthesis is limited (for reviews see Coulson 1980 and Dufossé et al. 2005)). In this respect, the fact that carotenoids are widely distributed in microorganisms including bacteria, yeast, fungus and algae offers a promising alternative for carotenoids production, reviewed recently in (Mata-Gómez et al. 2014). Furthermore, since bioreactors allow successful cultivation of different species of marine microorganisms, including diatoms, the latter are considered a good source of natural pigments. Accordingly, the most abundant pigment in diatoms, fucoxanthin, is available at present as a pure extract with important bioactivities (Mikami & Hosokawa 2013; Xia et al. 2013). Last but not least, it is worth mentioning that although a variety of metabolic engineering tools are available to manipulate the biosynthetic pathways, the production of some carotenoids is unachievable. This is due to the fact that our knowledge about the genes-encoding carotenoid biosynthetic enzymes in diatoms is still limited (Dambek et al. 2012).

In this paper, a method of extraction and purification of diatoxanthin and diadinoxanthin from *Phaeodactylum tricornutum* has been described. This unicellular alga is a cosmopolitan marine pennate diatom. Since both Diadino and Diato occur only in few algal groups including diatoms, these pigments might be considered as diatom-specific carotenoids. A four-step procedure including total pigment extraction, saponification, separation of carotenoids and open column chromatography enables to obtain all-*trans* isomers of Diadino and Diato of a purity of 99 % or more. It is worth to refer this method to the isolation of fucoxanthin and peridinin described in (Haugan et al. 1995).

## Materials and methods

### Reagents and chemicals

The organic solvents (ethyl acetate, methanol, acetonitrile, acetone, hexane isomers mix, diethyl ether, water) were of HPLC grade and purchased from Rathburn Chemicals Ltd. (Scotland) or Chromasolv (USA). The ammonium acetate *p.a.* was supplied by Avantor Performance Materials (Poland) and dissolved in HPLC grade water. For saponification and pigment partitioning, potassium hydroxide *p.a.* obtained from Avantor Performance Materials was used as well as rectified 95 % ethanol and extraction petroleum purchased from Polmos (Poland) and Avantor Performance Materials, respectively. Silica gel (Silica gel 60, pore size 0.040–0.063 mm (230–400 mesh ASTM)) purchased from Merck (Germany) were alkalised by hydrogen carbonate *p.a*. and washed by deionised water.

### HPLC analysis

Pigment fractions collected in each step of the procedure were dried under a stream of nitrogen, dissolved in extraction medium (methanol, 0.2 M ammonium acetate, ethyl acetate (81:9:10, *v*/*v*)) and analysed on a Nucleosil column (ET 250/8/4, 300–5, C18; Macherey & Nagel, Germany) by HPLC, applying a gradient according to Kraay et al. (1992). The elution was performed using solvent A (methanol, 0.5 M aqueous ammonium acetate, 85:15 *v*/*v*), solvent B (acetonitrile to water, 90:10 *v*/*v*) and solvent C (ethyl acetate) in a linear gradient (minutes/% solvent A/% solvent B/% solvent C): 0/60/40/0, 2/0/100/0, 7/0/80/20, 17/0/50/50, 21/0/30/70, 28.5/0/30/70, 29.5/0/100/0, 30.5/60/40/0, at a flow rate of 0.8 mL min^−1^. Reversed-phase HPLC was performed on an Agilent 1200 Series HPLC system equipped with a photodiode array detector and controlled by Agilent ChemStation software. Pigments were identified by comparing the on-line spectra with data from the literature and quantified using calibration curves calculated with standards, according to Lohr (2000). Absorption spectra were monitored at four wavelengths: 430, 440, 480 and 665 nm. The content (*n*/*V*_ex_) of each pigment was calculated by the following formula: ((*A*/*A*_c_/MW*1000)*V_ex_)/*V*_inj_ where *A* is the peak area, *A*_*c*_ is the peak area μg^−1^ pigment, MW is the molar weight, *V*_ex_ is the extract volume, *V*_inj_ is the injection volume and *n* is the number of moles.

### Culture conditions

**Fig. 1:**
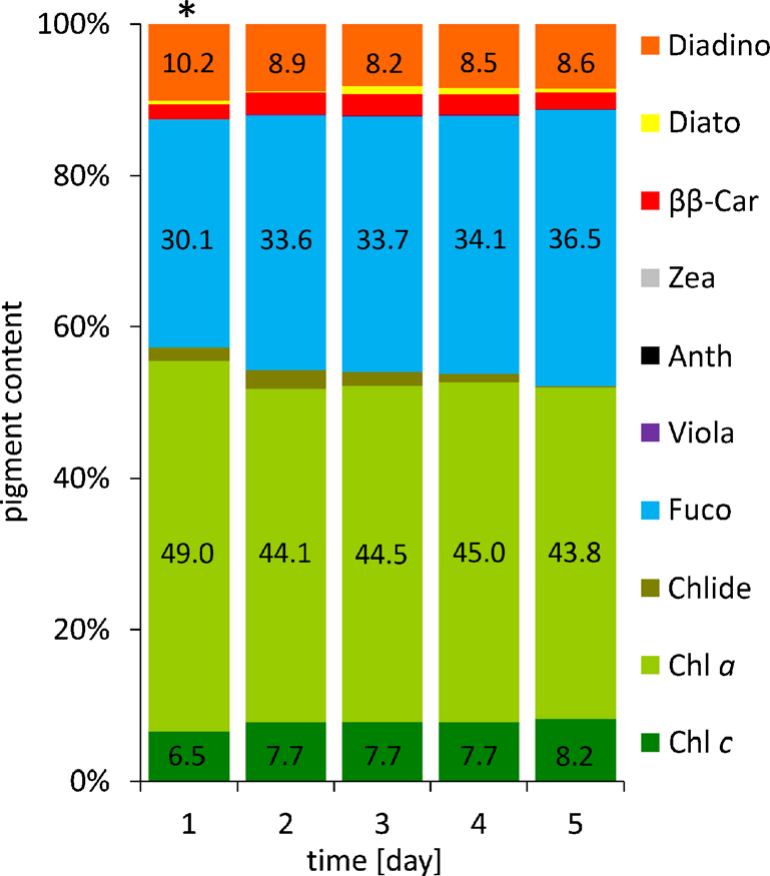
Dependence of pigment content on number of days of diatom culture. *Phaeodactylum tricornutum* was cultivated at 15 °C, under white fluorescent light (100 μmol photons m^−2^ s^−1^) in a 16/8 h day/night photoperiod. Samples were collected after 1 h of darkness. Pigment content was determined by HPLC and expressed as a percentage value. The data are means of three replicates; a sample marked with an asterisk is mean of seven replicates. Statistical data are given in Table A.1 in Supplementary Material

**Fig. 2:**
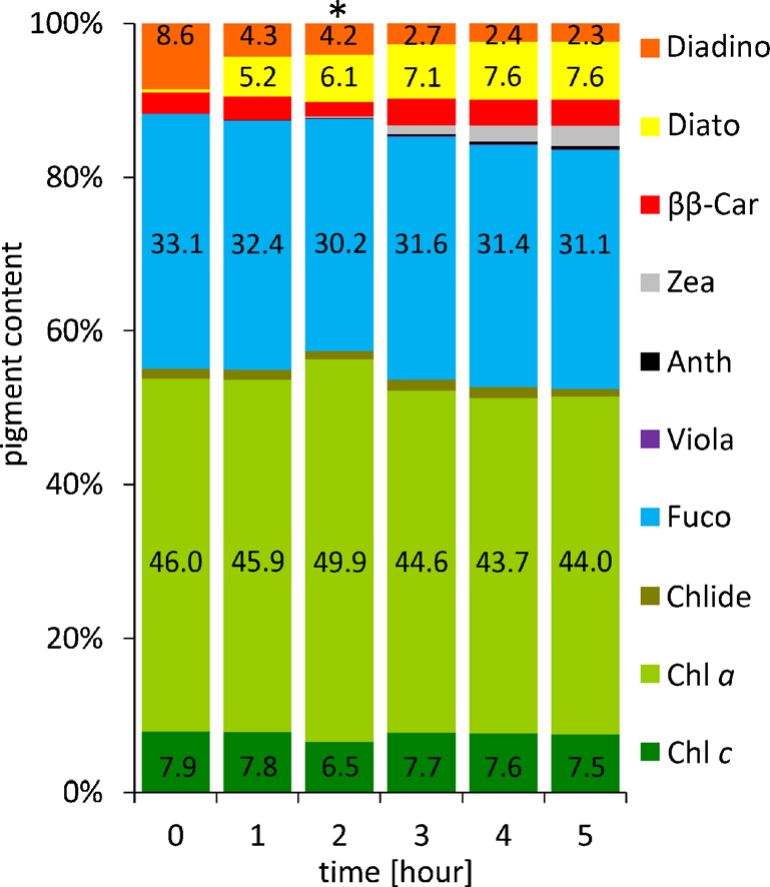
Pigment content in one-day diatom culture illuminated with 1250 μmol photons m^−2^ s^−1^ for 5 h. *Phaeodactylum tricornutum* was cultivated at 15 °C, under white fluorescent light (100 μmol photons m^−2^ s^−1^) in a 16/8 h day/night photoperiod. Samples were collected every hour. Pigment content was determined by HPLC and expressed as a percentage value. The data are means of three replicates; a sample marked with an asterisk is mean of seven replicates. Statistical data are given in Table A.1 in Supplementary Material

*Phaeodactylum tricornutum* Bohlin strain CCAP 1055/1 was obtained from the Culture Collection of Algae and Protozoa, UK. Cultures were grown in f/2-Si medium (Guillard 1975) made with artificial seawater supplemented with inorganic nutrients and vitamin mixture at 15 °C, under white light (absorption spectrum in A.1 in Supplementary Material A) of 100 μmol photons m^−2^ s^−1^ in a 16/8 h day/night photoperiod. Cells were inoculated every 3 or 4 days to maintain exponential phase of growth and reduce the number of dead cells. To provide optimal oxygen conditions, the volume of culture was lower than half of the flask volume and the number of cells was between 1 × 10^6^ to 3 × 10^6^ cells mL^−1^ which were counted microscopically in a Malassez chamber (Brand, Germany). The experiments were performed in flasks containing 1 L of diatom culture with initial density of 1 × 10^6^ cells mL^−1^, which were grown for 3 or 4 days to obtain density of approximately 3 × 10^6^ cells mL^−1^. A day before sample collection, the cells were inoculated into fresh medium to obtain 2 × 10^6^ cells mL^−1^. To examine the level of Diadino (results shown in Fig. 1), samples were collected for 5 days in light period, after 1 h of incubation in darkness. One-day-old culture was chosen as an appropriate for Diadino purification. To examine the level of Diato (results are shown in Fig. 2), cultures were illuminated with 1250 μmol photons m^−2^ s^−1^ white light for 5 h and samples were collected in every hour. Two-hour illumination was applied to Diato purification. In each case, the cells were harvested through centrifugation for 5 min at 8000×*g* at 4 °C. Pellet was frozen in liquid nitrogen and stored at −20 °C for no more than 1 week.

### Pigment extraction

Pigments were extracted from frozen cells through addition of extraction medium composed of methanol, 0.2 M ammonium acetate and ethyl acetate (81:9:10, *v*/*v*) based on Snoeijs et al. (2012), in a ratio of 10 mL per 2 × 10^9^ cells. The mixture was vortexed thoroughly and centrifuged for 4 min at 14000×*g* at 4 °C. Supernatants were collected and evaporated in an inert gas atmosphere to remove volatile compounds. To minimize the risk of destruction or undesired reactions, all manipulations during isolation and analysis should be carried out with some general precautions described in details in Rodriguez-Amaya (2001) and Schiedt & Liaaen-Jensen (1995) to avoid peroxide-containing ethers, strong light, room temperature and oxygen in air.

### Saponification and pigment partitioning

To semi-dried extract from 2 × 10^9^ cells ethanol was added up to 35 mL, followed by subsequent addition of aqueous potassium hydroxide (60 % *w*/*v*, 3.5 mL) (Goodwin 1955). Solution was mixed gently for 15–20 h at 4 °C. To separate chlorophylls from carotenoids, a volume of 40 mL hexane: diethyl ether (1:1 *v*/*v*), 10 mL extraction petroleum and 20 mL water were subsequently added, mixed gently and left to phase separate. Upper phase containing carotenoids was collected while lower phase was used to repeat the separation by using a volume of 10 mL hexane: diethyl ether (1:1 *v*/*v*), 2.5 mL extraction petroleum and 5 mL water. Then carotenoids fractions were combined and washed three times with 50 mL of water. To avoid formation of emulsions, gentle swirling was applied and final solution was transparent. It was evaporated in an inert gas atmosphere and stored at −20 °C for no more than 1 month.

### Open column chromatography

Silica gel was modified according to Nagy et al. (2009), namely by stirring with saturated aqueous sodium hydrogen carbonate solution for 2 h, filtration and washing with deionised water, then with acetone followed by drying under the hood. Modified gel was named silica-9 because of the pH of its aqueous suspension. An amount of 20 g of silica-9 was suspended in hexane: acetone (90:10 *v*/*v*), put in a 15-mm diameter glass column and applied to the chromatography of carotenoid fraction obtained from 2 × 10^9^ cells. Upon dissolving in approximately 3 mL hexane: acetone ratio (90:10 *v*/*v*), the sample was applied on the column and elution with hexane to acetone ratio (90:10 *v*/*v*) was carried out until the first fraction was collected. Then, the eluent was changed to hexane: acetone (80:20 *v*/*v*) and the second small fraction was observed and collected. The continued elution resulted in separation of further fraction, which was Diato. Once Diato was collected, the change of eluent to hexane: acetone (70:30 *v*/*v*) resulted in separation and collection of Diadino fraction. The last coloured band remaining on the top of the column was removed by acetone. After conditioning with hexane: acetone (90:10 *v*/*v*), the gel was reused. Collected samples of Diadino and Diato were evaporated in an inert gas atmosphere and stored at −80 °C.

### Statistical analysis

Each step of Diadino or Diato purification was performed in four replications which are represented by four individual diatom cultures constituting material to extraction of total pigments and subsequent purification steps. The level of pigments was expressed both as the percentage value and the absolute amount. The data shown in figures and tables are means of four replicates (three to seven replicates in Figs. 1 and 2). Statistical analysis included the determination of confidence intervals (CI) for a significance level (*P*) set to 0.05. Parameters such as standard deviation (SD), standard error (SE), significance level (P), lower limit of the confidence interval (CI _min_), upper limit of the confidence interval (CI _max_) are given in Supplementary materials (Table A.1).

## Results and discussion

### Culture conditions of diatoms

Regardless of the type of the xanthophyll cycle, the enzymatic removal of epoxy groups from xanthophylls occurs as a consequence of strong light treatment. On the other hand, light is necessary to ensure the elevated content of epoxidised xanthophylls in cells. Thus, special attention should be paid to the light conditions. Understandably, for these studies, diatoms were cultivated under high light, and depending on whether Diadino or Diato was to be collected, the cells were harvested in dark or light phase, respectively. Several culture conditions including illumination with white light of two intensities (1250 and 700 μmol photons m^−2^ s^−1^), for hours or days, in dark or light periods were tested. These preliminary studies have shown that while high levels of Diadino (up to 19 % of total pigments) and Diato (up to 17 % of total pigments) were obtained under a strong light, formation of *cis*-Diadino and *cis*-Diato and also an increase in Viola, Anth and Zea levels (A.3 in Supplementary Material) were observed, which made purification procedure even more difficult. Finally, white light of the intensity of 100 μmol photons m^−2^ s^−1^ in a 16/8 h day/night photoperiod was chosen as the most appropriate. Such medium light allowed a higher Diadino level (even 10 % (Fig. 1)) to be obtained compared to 7 % found in the cells growing under low light (30 μmol photons m^−2^ s^−1^) (A.3 in Supplementary Material). Concurrently, neither *cis* isomers nor the VAZ cycle pigment formation occurred (A.3 in Supplementary Material). Pigment content was measured during 5 days after 1 h of storage in darkness in the exponential phase of growth. Figure 1 shows the percentage values of each pigment found in sample extracts, including chlorophyll *a* (Chl *a*), chlorophyll *c*_*1*_ and *c*_*2*_ (Chl *c*), chlorophillide (Chlide), fucoxanthin (Fuco), β,β-carotene (ββ-Car), diadinoxanthin (Diadino), diatoxanthin (Diato), antheraxanthin (Anth), violaxanthin (Viola) and zeaxanthin (Zea). It is noteworthy that on the first day after inoculation, Diadino level was the highest and this time was chosen as an appropriate to collect sample. Aforementioned conditions have also been applied for the culture of *P. tricornutum* to obtain Diato with one exception, namely before collection cells were illuminated with white light of the intensity of 1250 μmol photons m^−2^ s^−1^ for no more than 2 h. This exposure time resulted in that Diato achieved the level of 6 % of the total pigment content. Although, continued illumination caused an increase in Diato content but evoked formation of Zea (Fig. 2). Here, it is worth pointing out that even small amounts of Viola, Anth and Zea, when present in pigment extract, are irremovable throughout the whole procedure, and final fractions of Diadino and Diato are contaminated with almost the same amounts of the VAZ cycle pigments as those found in the sample undergoing purification.

### Pigment extraction

The efficiency of pigment extraction were found to be dependent on two factors, namely the composition of extraction medium and the proportion between this solvent volume and the number of cells. Several kinds of extraction media have been tested by Snoeijs et al. (2012). Based on their studies, it was decided to use an extraction medium composed of methanol, ammonium acetate and ethyl acetate due to its high efficiency in Diadino extraction accompanied by low capability of Chl *a* and ββ-Car removal. Moreover, the presence of ammonium ions allows reduction in or even inhibition of isomerization of all-*trans* to *cis* forms of carotenoids. The most appropriate proportion between volume of extraction medium and the number of cells yielding the highest extraction recovery for Diadino and Diato was found to be of 10 mL per 2 × 10^9^ of diatom cells. To check the content of non-extracted pigments re-extraction was performed, which confirmed high efficiency of the first extraction (A.4 in Supplementary Material). Last but not least, it has been shown that gentle vortexing of suspended cells is sufficient and additional homogenization with glass beads has no impact on extraction efficiency (A.5 Supplementary Material).

### Saponification and pigment partitioning

**Fig. 3:**
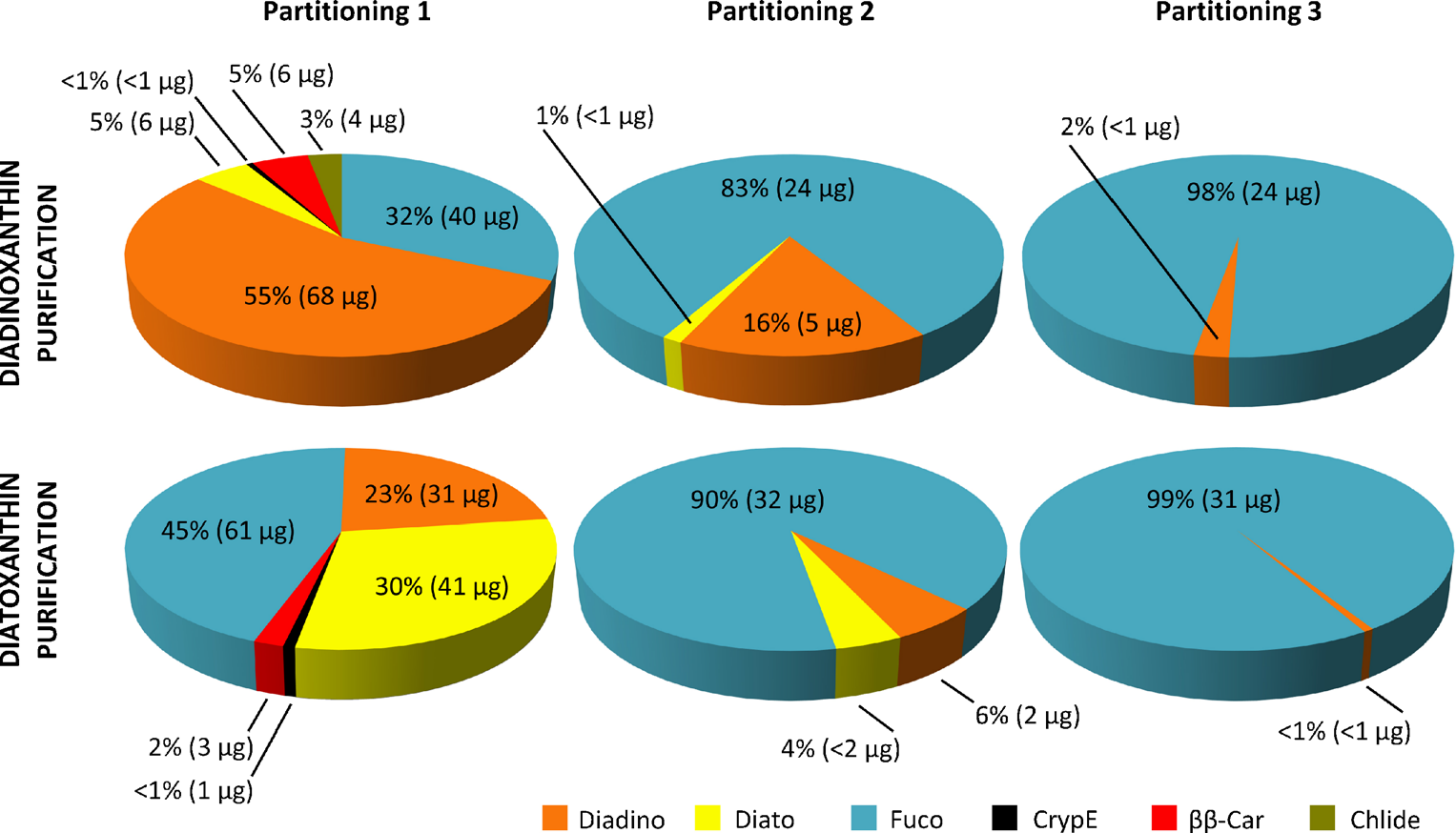
Pigment content of carotenoid fractions obtained for three consecutive partitioning subsequent to saponification in the diadinoxanthin and diatoxanthin purification procedure. Pigment content was determined by HPLC and expressed as a percentage value and as an absolute amount obtained from 2 × 10^9^ diatom cells. The data are means of four replicates. Statistical data are given in Table A.1 in Supplementary Material

**Fig. 4:**
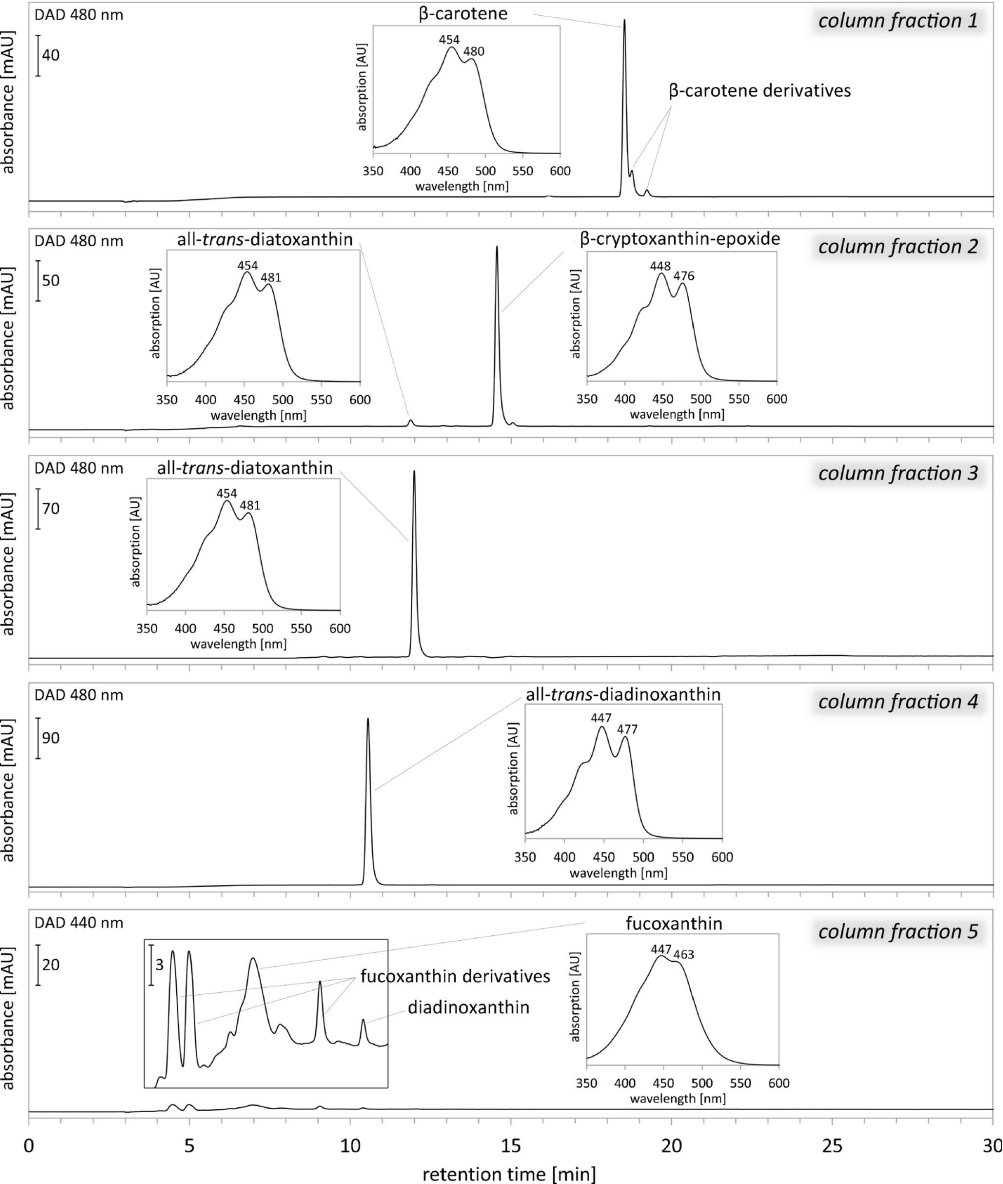
Chromatograms obtained with the method by Kraay and co-workers (see “Materials and methods” section) and absorption spectra recorded during HPLC-DAD analysis for consecutive carotenoid fractions collected from open column chromatography

Alkaline saponification of pigment extract and liquid-liquid partitioning by polar and non-polar solvents allow the removal chlorophylls and lipids, which upon saponification are easily separated from hydrophobic carotenoids. Since in *P. tricornutum* chlorophylls make up more than 50 % of pigments (Fig. 1), their removal substantially contributes to the enrichment of Diadino and Diato. Taking into account that some carotenoids including epoxy-carotenoids, i.e. Fuco, are unstable in alkali (Khachik 1986; Maoka 2016), saponification, in general, is not recommended. It is believed that the extent of degradation depends on the conditions used, being greater with higher concentration of alkali and on hot saponification (Kimura 1990). Saponification described in this paper resulted in Chl *a*, Fuco and ββ-Car degradation but had no impact on Diadino and Diato (see chromatograms of fractions eluted from column in Fig. 4). Then, partitioning carried out with the use of hexane: diethyl ether (1:1 *v*/*v*), extraction petroleum and water in proportion 4:1:2 (*v*/*v*) gave great separation result. The use of hexane and diethyl ether was found not to be sufficient, and as a result, no partitioning was observed (A.6 in Supplementary Material). The effect of the addition of petroleum was only slight, and finally, combination of all four components could provide the excellent partitioning results with sharp phase boundaries (A.6 Supplementary Material). Three repetitions of partitioning were performed but single steps allowed to recover most of Diadino (68 μg) or Diato (41 μg) and left only 5 or 1.5 μg, respectively (Fig. 3). In the second and third partitioning, Fuco was the dominant pigment, 83 % or even 99 % (Fig. 3). The content of Diadino or Diato in second partitioning was 16 or 4 %, respectively and therefore it seems reasonable to ignore these steps.

Combined carotenoid fractions obtained from partitioning subsequent to saponification are to be subjected to washing with distilled water to remove alkali. While agitation of the mixture is typically recommended, it was found to lead to the formation of an emulsion which was difficult to break and resulted in the loss of carotenoids to the aqueous phase. Instead, gentle swirling reached expectations and upon three repetitions of washing, a transparent solution was obtained (A.7 in Supplementary Material).

### Open column chromatography

Open column chromatography is widely used as a method of choice to separate and purify photosynthetic pigments. However, calcium carbonate, alumina, magnesium oxide etc., which are very efficient and do not cause decomposition of carotenoids, are characterized by very small capacity. The latter makes them practically unfavourable when considering preparative scale chromatography. On the other hand, silica gels of high capacities, due to their acidic character are not suitable for carotenoids (Bernhard 1995; Nagy et al. 2009). To overcome this problem, eluent is often enriched with triethylamine; however, the base is then difficult to remove as well as changes in the retention occur. Therefore, following the idea of Nagy et al. (2009), modified silica gel, which is chemically converted to a basic form and retains high capacity, was used. Application of silica-9 and gradient elution with hexane and acetone to separate Diato and Diadino from other carotenoids and also from each other was proved to be efficient. Moreover, it induced no transformation or decomposition of the DD cycle pigments. It should be noticed that the use of *n*-hexane instead of hexane mix of isomers in this chromatography completely stops migration from the second fraction. The steps of chromatographic separation are described in Table 1.

**Table 1:** The consecutive steps of separation by open column chromatography using silica-9 as a stationary phase. Approximate volumes of eluents and runtime are given for the following conditions: (i) 20 g of dry silica-9 suspended in hexane: acetone (90:10 *v*/*v*) was applied to the column; (ii) glass chromatography column with filter disc, 15 mm; (iii) evaporated carotenoid fraction after saponification of pigment extract obtained from 2 × 10^9^ cells was dissolved in hexane: acetone (90:10 *v*/*v*) and applied to the column; (iv) chromatography was carried out at room temperature

Eluent	Description	Product
Hexane: acetone (90:10 *v*/*v*)	Clearly separated band migrates down through the column for approximately 25 min and approximately 40 mL of eluent is used. Three to four millilitres of this fraction is collected.	β,β-Carotene and its derivatives
Hexane: acetone (80:20 *v*/*v*)	After the change of eluent, a hardly visible band (more intensive in the case of Dtx purification) migrates for about 30 min and approximately 50 mL of eluent is used. A volume of 6–8 mL of this fraction is collected.	Cryptoxanthin-epoxide
	Third band is very intense and in half-length of column, it splits into two bands, which become better separated with time. From the second fraction, migration of the third one takes about 50 min and approximately 80 mL of eluent is used. A volume of 14–20 mL of this fraction is collected; in the case of Dtx purification, their colour is very intensive.	Diatoxanthin
Hexane: acetone (70:30 *v*/*v*)	Clearly marked band migrates for about 20 min since Dtx is collected and approximately 40 mL of eluent is used. A volume of 15–22 mL of this fraction is collected.	Diadinoxanthin
Acetone	Acetone results in migration of all remaining fractions, which are visible as four blurred bands collected together in a volume of 17–25 mL. Their migration takes about 30 min and approximately 60 mL of eluent is used.	Fucoxanthin derivatives

**Fig. 5:**
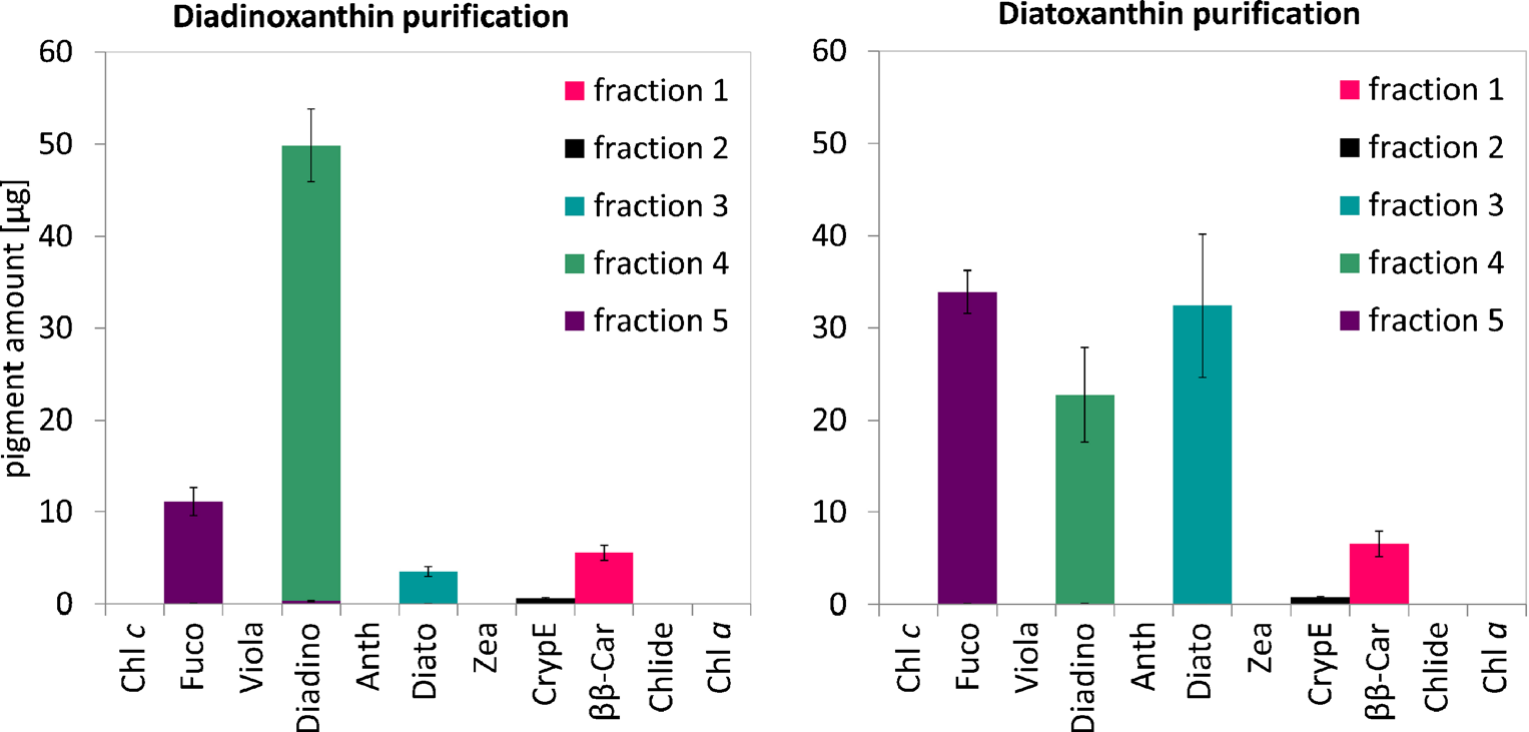
The amount of pigments in each fraction collected from the column which was obtained at the end of the purification process of pigments extracted from 2 × 10^9^ cells. Pigment content was determined by HPLC and expressed as an absolute amount. The *error bars* represent standard deviation (SD). The data are means of four replicates. Statistical data are given in Table A.1 in Supplementary Material

**Fig. 6:**
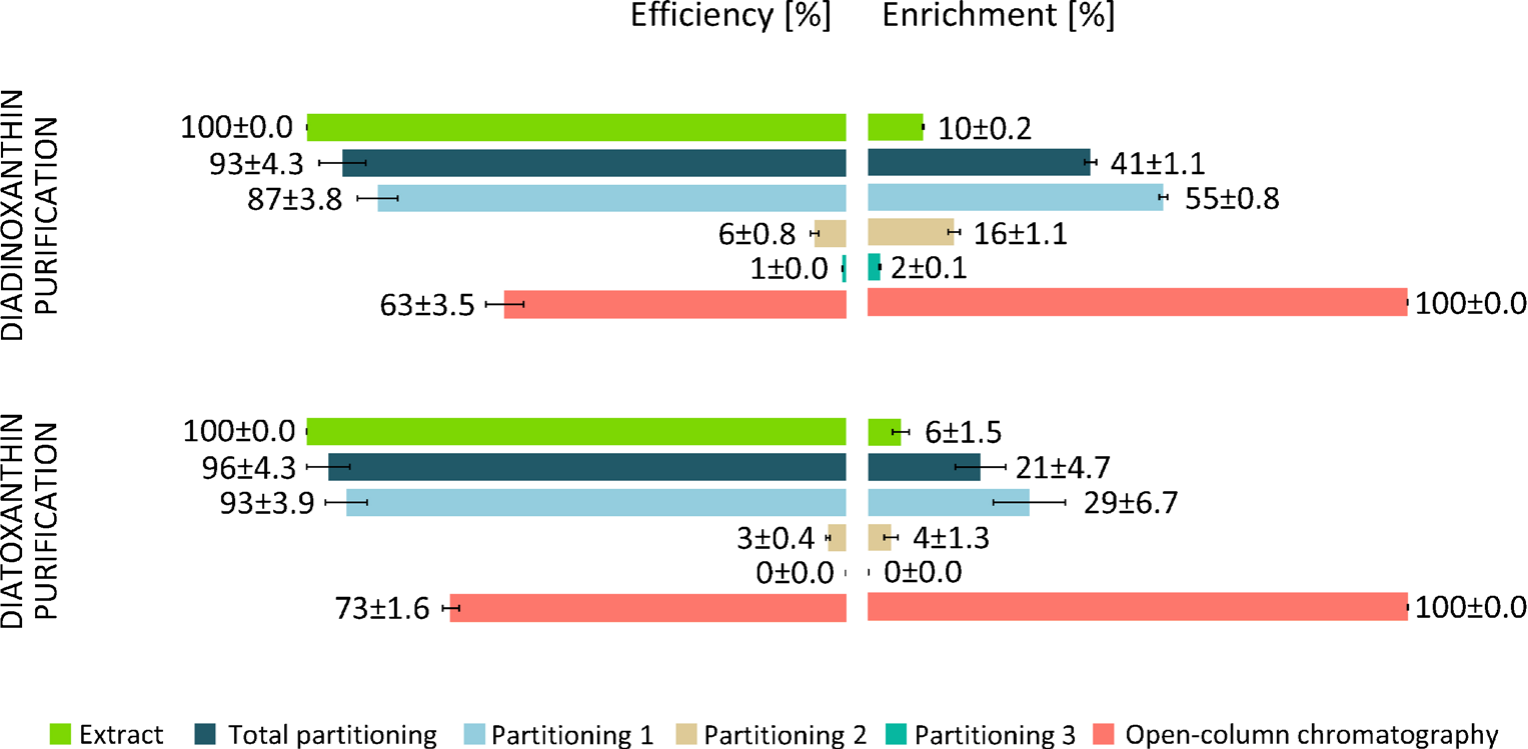
Efficiency (*left side*) and enrichment (*right side*) of diadinoxanthin and diatoxanthin purification in each step of the procedure. Partitioning 1, 2 and 3 represent first, second and third carotenoid fractions; total partitioning represents total yield estimated for three combined carotenoid fractions subjected to separation by partition. Pigment content was determined by HPLC and expressed as a percentage. The *error bars* represent standard deviation (SD). The data are means of four replicates. Statistical data are given in Table A.1 in Supplementary Material

Each fraction collected from the open column chromatography was analysed by HPLC (Fig. 4). Chromatogram recorded for the first fraction consisted of three peaks identified as ββ-Car and its derivatives (Fig. 5) in an amount of 5.5 to 6.5 μg (Fig. 6). The second fraction included mainly CrypE (89 % in Diadino or 91 % in Diato purification) and low amount of Diato and Fuco derivatives (Figs. 5 and 6). The third fraction was all-*trans* Diato, with its amount in samples intended for Diadino purification of approximately 4 μg and much higher (32 μg) in the case of Diato purification (Figs. 5 and 6). The fourth fraction corresponded to all-*trans* Diadino in the amount of 23 or 50 μg during Diato or Diadino purification, respectively (Figs. 5 and 6). The fifth fraction included traces of Fuco derivatives (11 to 34 μg) and Diadino (less than 0.3 μg) (Figs. 5 and 6). Elution of the fifth fraction is not necessary; however, after its removal, the silica gel might be reused. The purity of Diadino and Diato was estimated to be of 99 % or more. The amounts of purified pigments obtained from 2 × 10^9^ diatom cells were 2.44 × 10^−7^ and 1.16 × 10^−7^ g for Diadino and Diato, respectively.

### Efficiency of diatoxanthin and diadinoxanthin purification

The efficiency of each step in the Diadino or Diato purification procedure was estimated based on the amount of collected pigments and it is shown in Fig. 6. While the pigment extract included 10 % of Diadino and 6 % of Diato, separation by partition resulted in the significant enrichment of Diadino (41 %) and Diato (21 %) and only slight loss of this pigment was noted. This yielded the efficiency of this step at the very high level of approximately 90 % (Fig. 6). However, the first partitioning was the most efficient whereas the second and the third ones increased this value only of 6 and 3 % for Diadino and Diato, respectively. Thus, taking into account the time and chemical cost, the repetitions might be skipped. The open column chromatography resulted in the most pronounced loss of pigment. Final efficiency of described purification procedure was estimated to be 63 % for Diadino and 73 % for Diato.

## Conclusions

In this paper, a method of all-*trans* diatoxanthin and all-*trans* diadinoxanthin purification from marine diatom *P. tricornutum* has been described. The protocol consists of four steps including pigment extraction, saponification, separation by partition and open column chromatography. Conditions of diatom culture were optimized paying special attention to the light to produce high content of Ddx and Dtx and concurrently to avoid the formation of their *cis* isomers as well as the VAZ cycle pigments. Extraction medium consisted of methanol, ammonium acetate and ethyl acetate which ensured efficient extraction of Ddx and Dtx from diatom cells. By use of the four component mixture with hexane: diethyl ether (1:1 *v*/*v*), petroleum and water in proportion 4:1:2 (*v*/*v*), an excellent separation of chlorophylls and carotenoids was achieved, which contributed to the significant enrichment of Ddx and Dtx. Finally, by open column chromatography with alkalised silica gel, Ddx and Dtx were collected of a purity of 99 % or more. Final efficiency of our purification procedure was estimated to be 63 % for Ddx and 73 % for Dtx and enabled to get about 30–50 μg of these xanthophylls from 1 L of diatom culture with density of 2 × 10^6^ cells mL^−1^. This procedure is dedicated to both analytical and preparative scale. This is of special importance given that until now neither Ddx nor Dtx were commercially available in amounts greater than those used as HPLC standards.

## Electronic supplementary material

ESM 1 (DOCX 1014 kb)
