# Supplementary material for: Isolation and purification of all-trans diadinoxanthin and all-trans diatoxanthin from diatom Phaeodactylum tricornutum
Source: J Appl Phycol. 2016 Sep 27;29(1):79–87. doi: 10.1007/s10811-016-0961-x (PMC5346133; doi:10.1007/s10811-016-0961-x)
Supplement: Supplementary file 1 — (DOCX 1014 kb) [file 10811_2016_961_MOESM1_ESM.docx]

**Isolation and purification of all-*trans* diadinoxanthin and all-*trans* diatoxanthin from diatom *Phaeodactylum tricornutum***

**Journal of Applied Phycology**

Paulina Kuczynska^1,🖂^ & Malgorzata Jemiola-Rzeminska^1,2^

^1^ Department of Plant Physiology and Biochemistry, Faculty of Biochemistry, Biophysics and Biotechnology, Jagiellonian University, Krakow, Poland

^2^ Malopolska Centre of Biotechnology, Krakow, Poland

^🖂^ kuczynska.paul@gmail.com

**Appendix A: Supporting information**

**
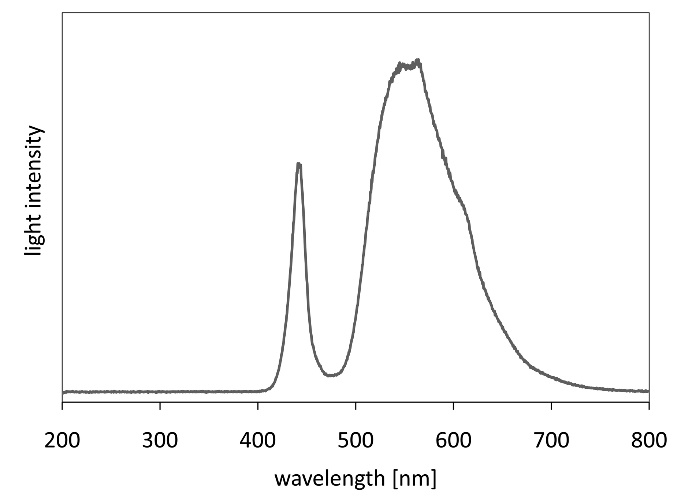
**

**A.1** LED panel light spectrum recorded for the diatom growing chamber


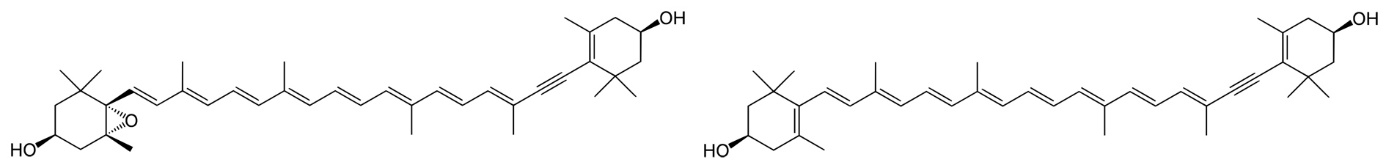


**A.2** Structural formula of all-*trans* diadinoxanthin (left-side) and all-*trans* diatoxanthin (right-side)


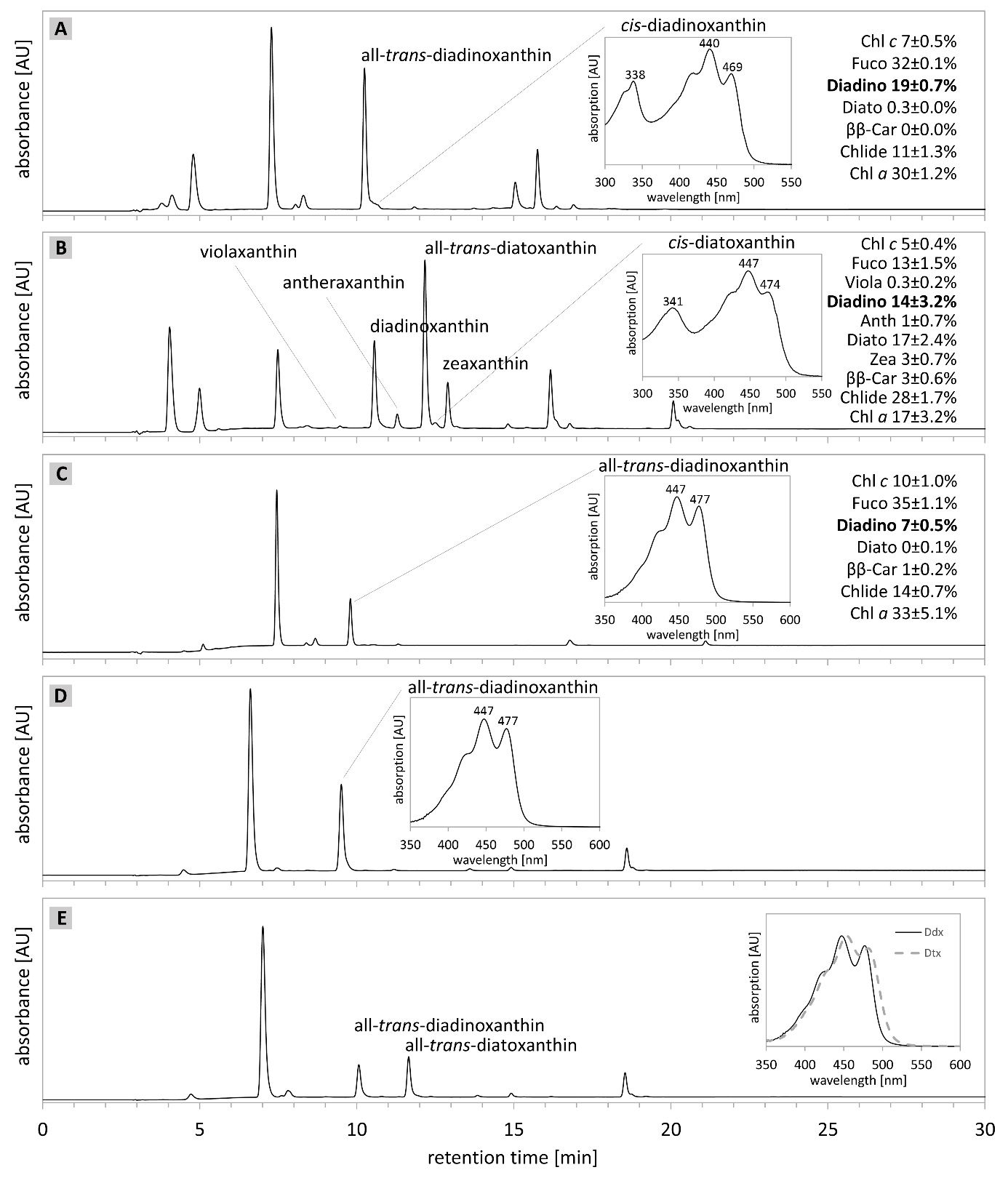


**A.3** Chromatograms of pigment extracts recorded for *P. tricornutum* cultivated at 15°C, under white light of the intensity of: **a)** 700 μmol photons m^-2^s^-1^ in a 6/18 h day/night photoperiod; high content of Diadino but also its *cis* isomer were identified; **b)** 1250 μmol photons m^-2^s^-1^, contains high Diato level but also *cis*-Diato, Viola, Anth and Zea; **c)** 30 μmol photons m^-2^s^-1^ in a 16/8 h day/night photoperiod; Diadino level was low; **d)** 100 μmol photons m^-2^s^-1^) in a 16/8 h day/night photoperiod; samples were collected after 1 h of darkness and used for Diadino purification; **e)** 100 μmol photons m^-2^s^-1^ in a 16/8 h day/night photoperiod; one-day culture was illuminated (1250 μmol photons m^-2^s^-1^) for 5 h and used for Diato purification. Inserts show the absorption spectra recorded during HPLC-DAD analysis (for details see the text). Panels A, B and C include pigment content determined by HPLC and expressed as a percentage value. Pigment content in samples presented in panels D and E were given in Fig. 1 and Fig. 2 (section 3.1)


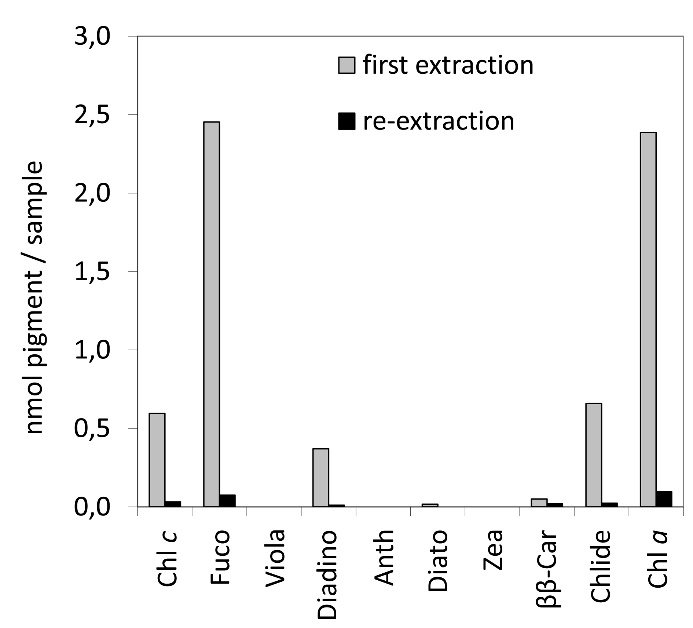


**A.4** Results of quantitative HPLC analysis (for details see Material & Methods) of pigment content of *P. tricornutum* obtained by extraction and re-extraction with the same extraction medium


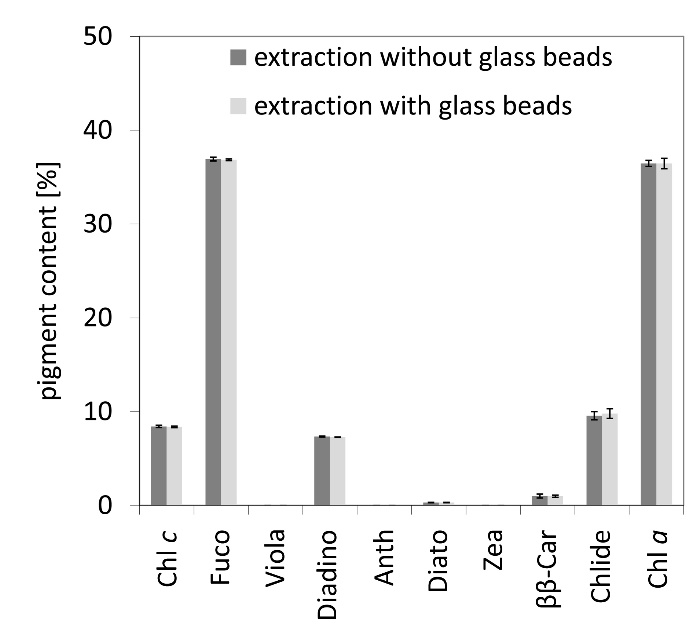


**A.5** Comparative data of quantitative HPLC analysis (for details see Material & Methods) of pigment content of *P. tricornutum* obtained by simple extraction with extraction medium and extraction supported by homogenization by glass-bead agitation


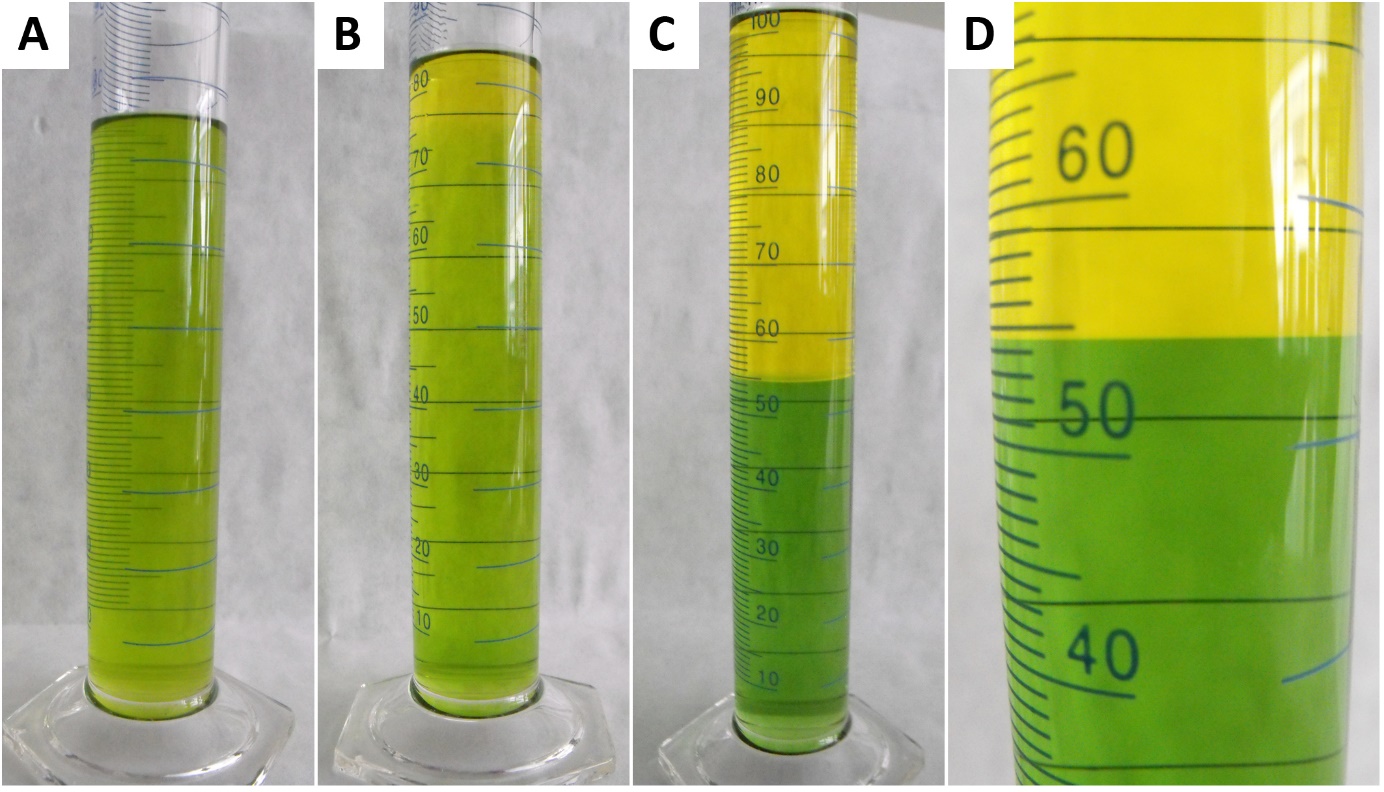


**A.6** Photographs of pigment partitioning (subsequent to saponification) with **a)** hexane:diethyl ether (1:1 v/v), **b)** hexane:diethyl ether (1:1 v/v) and extraction petroleum, **c)** hexane:diethyl ether (1:1 v/v), extraction petroleum and water, **d)** close up of photo. c


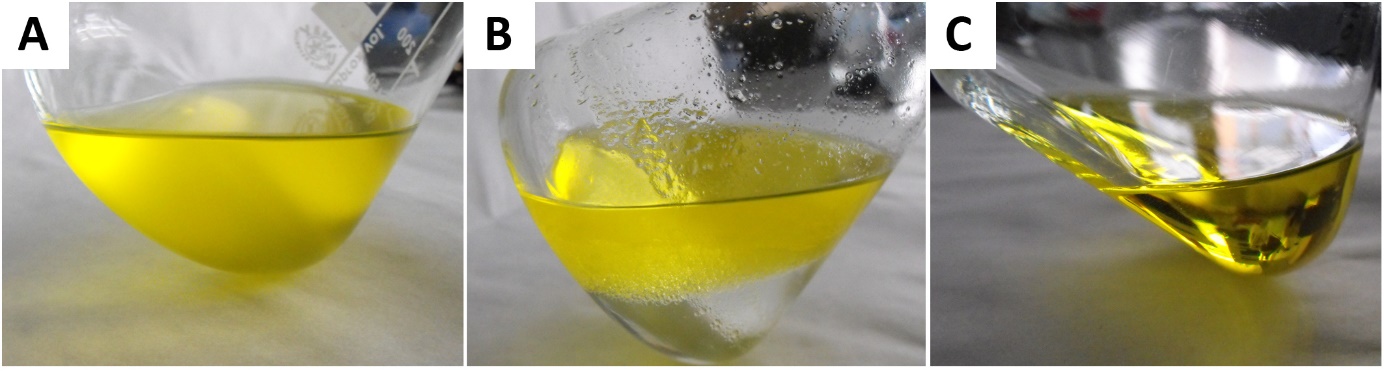


**A.7** Photographs of washing procedure of the carotenoid fraction (subsequent to saponification and partitioning). **a)** turbid solution before washing, **b)** intermediate step accompanying alkali removal during the first and second washing cycles, **c)** transparent solution obtained after the third washing with distilled water

**Table A.1** Statistical data of samples in Figures 1, 2, 3 and 6 including number of replications (N), mean (M), standard deviation (SD), standard error (SE), significance level (P), lower limit of the confidence interval (CI _min_), upper limit of the confidence interval (CI _max_)

| **sample** | **pigment** | **N** | **M** | **SD** | **SE** | **P** | **CI _min_** | **CI _max_** |
| --- | --- | --- | --- | --- | --- | --- | --- | --- |
| **Fig. 1** Dependence of pigment content on number of days of diatom culture. *Phaeodactylum tricornutum* was cultivated at 15°C, under white fluorescent light (100 μmol photons m^-2^s^-1^) in a 16/8 h day/night photoperiod. Samples were collected after 1 h of darkness. Pigment content was determined by HPLC and expressed as a percentage value | | | | | | | | |
| Day 1 | Chl *c*  Fuco  Diadino  Diato  ββ-Car  Chlide  Chl *a* | 7  7  7  7  7  7  7 | 6,49  30,14  10,15  0,49  1,92  1,77  49,04 | 1,41  3,61  0,87  0,40  0,76  1,03  6,27 | 0,53  1,36  0,33  0,15  0,29  0,39  2,37 | 0,05  0,05  0,05  0,05  0,05  0,05  0,05 | 5,18  26,80  9,35  0,11  1,22  0,82  43,24 | 7,80  33,47  10,96  0,86  2,63  2,72  54,84 |
| Day 2 | Chl *c*  Fuco  Diadino  Diato  ββ-Car  Chlide  Chl *a* | 3  3  3  3  3  3  3 | 7,69  33,64  8,89  0,19  2,88  2,51  44,10 | 0,18  0,49  0,88  0,07  0,09  0,11  0,46 | 0,10  0,28  0,51  0,04  0,05  0,06  0,26 | 0,05  0,05  0,05  0,05  0,05  0,05  0,05 | 7,37  32,74  7,28  0,06  2,70  2,30  43,26 | 8,02  34,54  10,50  0,33  3,05  2,71  44,93 |
| Day 3 | Chl *c*  Fuco  Diadino  Diato  ββ-Car  Chlide  Chl *a* | 3  3  3  3  3  3  3 | 7,73  33,74  8,21  1,06  2,80  1,83  44,49 | 0,16  0,68  0,21  0,76  0,06  0,25  0,61 | 0,09  0,39  0,12  0,44  0,03  0,15  0,35 | 0,05  0,05  0,05  0,05  0,05  0,05  0,05 | 7,44  32,50  7,83  0,00  2,69  1,37  43,36 | 8,02  34,99  8,59  2,45  2,91  2,30  45,61 |
| Day 4 | Chl *c*  Fuco  Diadino  Diato  ββ-Car  Chlide  Chl *a* | 3  3  3  3  3  3  3 | 7,71  34,07  8,47  0,84  2,68  1,14  44,97 | 0,08  0,28  0,60  0,29  0,05  0,05  0,47 | 0,05  0,16  0,34  0,17  0,03  0,03  0,27 | 0,05  0,05  0,05  0,05  0,05  0,05  0,05 | 7,55  33,56  7,38  0,30  2,58  1,04  44,11 | 7,86  34,57  9,57  1,37  2,77  1,24  45,83 |
| Day 5 | Chl *c*  Fuco  Diadino  Diato  ββ-Car  Chlide  Chl *a* | 3  3  3  3  3  3  3 | 8,16  36,45  8,57  0,51  2,23  0,17  43,82 | 0,12  0,28  0,53  0,24  0,08  0,03  0,49 | 0,07  0,16  0,30  0,14  0,05  0,02  0,28 | 0,05  0,05  0,05  0,05  0,05  0,05  0,05 | 7,95  35,94  7,60  0,07  2,09  0,11  42,93 | 8,38  36,97  9,53  0,95  2,38  0,23  44,72 |
| **Fig. 2** Pigment content in one-day diatom culture illuminated with light of intensity of 1250 μmol photons m^-2^s^-1^ for 5 h. *P. tricornutum* was cultivated at 15°C, under white fluorescent light (100 μmol photons m^-2^s^-1^) in a 16/8 h day/night photoperiod. Samples were collected in every hour. Pigment content was determined by HPLC and expressed as a percentage value | | | | | | | | |
| hour 0 | Chl *c*  Fuco  Viola  Diadino  Anth  Diato  Zea  ββ-Car  Chlide  Chl *a* | 3  3  3  3  3  3  3  3  3  3 | 7,86  33,09  0,00  8,64  0,00  0,40  0,00  2,78  1,24  45,99 | 0,03  0,06  0,00  0,02  0,00  0,00  0,00  0,01  0,07  0,14 | 0,02  0,03  0,00  0,01  0,00  0,00  0,00  0,00  0,04  0,08 | 0,05  0,05  0,05  0,05  0,05  0,05  0,05  0,05  0,05  0,05 | 7,81  32,99  0,00  8,60  0,00  0,39  0,00  2,77  1,11  45,73 | 7,91  33,19  0,00  8,68  0,00  0,40  0,00  2,79  1,38  46,26 |
| hour 1 | Chl *c*  Fuco  Viola  Diadino  Anth  Diato  Zea  ββ-Car  Chlide  Chl *a* | 3  3  3  3  3  3  3  3  3  3 | 7,78  32,41  0,03  4,34  0,04  5,20  0,00  2,96  1,35  45,88 | 0,04  0,04  0,05  0,33  0,04  0,36  0,00  0,05  0,05  0,07 | 0,02  0,02  0,03  0,19  0,02  0,21  0,00  0,03  0,03  0,04 | 0,05  0,05  0,05  0,05  0,05  0,05  0,05  0,05  0,05  0,05 | 7,72  32,34  0,00  3,73  0,00  4,53  0,00  2,87  1,25  45,75 | 7,85  32,48  0,11  4,95  0,11  5,87  0,00  3,05  1,44  46,02 |
| hour 2 | Chl *c*  Fuco  Viola  Diadino  Anth  Diato  Zea  ββ-Car  Chlide  Chl *a* | 7  7  7  7  7  7  7  7  7  7 | 6,49  30,23  0,04  4,15  0,06  6,11  0,14  1,82  1,08  49,87 | 1,28  1,80  0,05  0,89  0,08  1,11  0,18  0,94  0,61  4,37 | 0,48  0,68  0,02  0,33  0,03  0,42  0,07  0,35  0,23  1,65 | 0,05  0,05  0,05  0,05  0,05  0,05  0,05  0,05  0,05  0,05 | 5,30  28,57  0,00  3,33  0,00  5,08  0,00  0,96  0,52  45,83 | 7,67  31,89  0,09  4,97  0,14  7,13  0,31  2,69  1,65  53,92 |
| hour 3 | Chl *c*  Fuco  Viola  Diadino  Anth  Diato  Zea  ββ-Car  Chlide  Chl *a* | 3  3  3  3  3  3  3  3  3  3 | 7,67  31,64  0,10  2,74  0,24  7,09  1,14  3,37  1,41  44,60 | 0,05  0,14  0,01  0,32  0,05  0,28  0,02  0,18  0,13  0,17 | 0,03  0,08  0,01  0,19  0,03  0,16  0,01  0,11  0,08  0,10 | 0,05  0,05  0,05  0,05  0,05  0,05  0,05  0,05  0,05  0,05 | 7,59  31,38  0,08  2,15  0,15  6,57  1,10  3,03  1,17  44,28 | 7,76  31,90  0,12  3,33  0,34  7,61  1,19  3,71  1,65  44,91 |
| hour 4 | Chl *c*  Fuco  Viola  Diadino  Anth  Diato  Zea  ββ-Car  Chlide  Chl *a* | 3  3  3  3  3  3  3  3  3  3 | 7,58  31,44  0,10  2,37  0,35  7,57  2,08  3,30  1,53  43,68 | 0,05  0,11  0,00  0,24  0,06  0,19  0,05  0,15  0,03  0,08 | 0,03  0,06  0,00  0,14  0,04  0,11  0,03  0,09  0,02  0,05 | 0,05  0,05  0,05  0,05  0,05  0,05  0,05  0,05  0,05  0,05 | 7,49  31,24  0,10  1,92  0,24  7,23  1,99  3,02  1,47  43,53 | 7,67  31,64  0,11  2,82  0,46  7,92  2,17  3,57  1,59  43,83 |
| hour 5 | Chl *c*  Fuco  Viola  Diadino  Anth  Diato  Zea  ββ-Car  Chlide  Chl *a* | 3  3  3  3  3  3  3  3  3  3 | 7,48  31,07  0,11  2,34  0,46  7,59  2,60  3,33  0,99  44,03 | 0,02  0,13  0,00  0,28  0,08  0,14  0,07  0,24  0,62  0,94 | 0,01  0,08  0,00  0,16  0,05  0,08  0,04  0,14  0,36  0,54 | 0,05  0,05  0,05  0,05  0,05  0,05  0,05  0,05  0,05  0,05 | 7,44  30,82  0,10  1,84  0,31  7,34  2,47  2,89  0,00  42,30 | 7,51  31,32  0,11  2,85  0,61  7,84  2,73  3,77  2,13  45,76 |
| **Fig. 3** Pigment content of carotenoid fractions obtained for three consecutive partitioning subsequent to saponification in diadinoxanthin and diatoxanthin purification procedure. Pigment content was determined by HPLC and expressed as a percentage value and as an absolute amount obtained from 2x10^9^ diatom cells. | | | | | | | | |
| Diadinoxanthin purification | | | | | | | | |
| Partitioning 1 | Fuco  Diadino  Diato  CrypE  ββ-Car  Chlide | 4  4  4  4  4  4 | 31,72  54,76  4,93  0,57  4,99  3,01 | 0,88  0,77  0,08  0,03  0,42  1,14 | 0,44  0,38  0,04  0,01  0,21  0,57 | 0,05  0,05  0,05  0,05  0,05  0,05 | 30,33  53,54  4,80  0,53  4,32  1,19 | 33,11  55,98  5,07  0,61  5,67  4,83 |
| Partitioning 2 | Fuco  Diadino  Diato | 4  4  4 | 82,75  15,97  1,28 | 1,16  1,09  0,09 | 0,58  0,55  0,04 | 0,05  0,05  0,05 | 80,91  14,23  1,14 | 84,60  17,71  1,42 |
| Partitioning 3 | Fuco  Diadino | 4  4 | 97,81  2,19 | 0,14  0,14 | 0,07  0,07 | 0,05  0,05 | 97,60  1,97 | 98,03  2,40 |
| Diatoxanthin purification | | | | | | | | |
| Partitioning 1 | Fuco  Diadino  Diato  CrypE  ββ-Car | 4  4  4  4  4 | 44,78  22,65  29,90  0,75  1,92 | 1,90  5,51  6,70  0,07  0,45 | 0,95  2,76  3,35  0,04  0,22 | 0,05  0,05  0,05  0,05  0,05 | 41,76  13,88  19,25  0,63  1,21 | 47,80  31,42  40,55  0,86  2,63 |
| Partitioning 2 | Fuco  Diadino  Diato | 4  4  4 | 90,05  5,72  4,23 | 0,50  1,09  1,28 | 0,25  0,54  0,64 | 0,05  0,05  0,05 | 89,25  3,99  2,19 | 90,85  7,45  6,27 |
| Partitioning 3 | Fuco  Diadino | 4  4 | 99,37  0,63 | 0,44  0,44 | 0,22  0,22 | 0,05 | 98,67  0,00 | 100,00  1,33 |
| **Fig. 5** The amount of pigments in each fraction collected from the column which was obtained at the end of purification process of pigments extracted from 2x10^9^ cells. Pigment content was determined by HPLC and expressed as an absolute amount. | | | | | | | | |
| Diadinoxanthin purification | | | | | | | | |
| Fraction 1 | ββ-Car | 4 | 5,53 | 0,81 | 0,41 | 0,05 | 4,24 | 6,83 |
| Fraction 2 | Fuco  Diato  CrypE | 4  4  4 | 0,05  0,02  0,60 | 0,02  0,01  0,10 | 0,01  0,00  0,05 | 0,05  0,05  0,05 | 0,03  0,01  0,45 | 0,08  0,03  0,76 |
| Fraction 3 | Diato | 4 | 3,56 | 0,54 | 0,27 | 0,05 | 2,70 | 4,41 |
| Fraction 4 | Diadino | 4 | 49,85 | 3,93 | 1,96 | 0,05 | 43,60 | 56,10 |
| Fraction 5 | Fuco  Diadino | 4  4 | 11,13  0,31 | 1,53  0,08 | 0,77  0,04 | 0,05  0,05 | 8,69  0,19 | 13,57  0,43 |
| Diatoxanthin purification | | | | | | | | |
| Fraction 1 | ββ-Car | 4 | 6,54 | 1,41 | 0,70 | 0,05 | 4,30 | 8,77 |
| Fraction 2 | Fuco  Diato  CrypE | 4  4  4 | 0,07  0,01  0,76 | 0,04  0,00  0,10 | 0,02  0,00  0,05 | 0,05  0,05  0,05 | 0,01  0,00  0,59 | 0,13  0,01  0,92 |
| Fraction 3 | Diato | 4 | 32,44 | 7,77 | 3,88 | 0,05 | 20,08 | 44,80 |
| Fraction 4 | Diadino | 4 | 22,73 | 5,12 | 2,56 | 0,05 | 14,59 | 30,87 |
| Fraction 5 | Fuco  Diadino | 4  4 | 33,89  0,04 | 2,35  0,08 | 1,18  0,04 | 0,05  0,05 | 30,14  0,00 | 37,63  0,16 |
| **Fig. 6** Efficiency (left-side) and enrichment (right-side) of diadinoxanthin and diatoxanthin purification in each step of the procedure. Partitioning 1,2 and 3 represent first, second and third carotenoid fractions, total partitioning represents total yield estimated for three combined carotenoid fractions subjected to separation by partition. Pigment content was determined by HPLC and expressed as a percentage value. | | | | | | | | |
| Diadinoxanthin purification | | | | | | | | |
| Efficiency | | | | | | | | |
| Extract | Ddx | 4 | 100,00 | 0,00 | 0,00 | 0,05 | 100,00 | 100,00 |
| Total partitioning | Ddx | 4 | 93,40 | 4,31 | 2,15 | 0,05 | 86,54 | 100,00 |
| Partitioning 1 | Ddx | 4 | 86,87 | 3,76 | 1,88 | 0,05 | 80,89 | 92,85 |
| Partitioning 2 | Ddx | 4 | 5,85 | 0,76 | 0,38 | 0,05 | 4,65 | 7,05 |
| Partitioning 3 | Ddx | 4 | 0,68 | 0,04 | 0,02 | 0,05 | 0,62 | 0,74 |
| Chromatography | Ddx | 4 | 63,32 | 3,53 | 1,76 | 0,05 | 57,70 | 68,93 |
| Enrichment | | | | | | | | |
| Extract | Ddx | 4 | 10,23 | 0,15 | 0,07 | 0,05 | 10,00 | 10,46 |
| Total partitioning | Ddx | 4 | 41,22 | 1,09 | 0,55 | 0,05 | 39,48 | 42,96 |
| Partitioning 1 | Ddx | 4 | 54,76 | 0,77 | 0,38 | 0,05 | 53,54 | 55,98 |
| Partitioning 2 | Ddx | 4 | 15,97 | 1,09 | 0,55 | 0,05 | 14,23 | 17,71 |
| Partitioning 3 | Ddx | 4 | 2,19 | 0,14 | 0,07 | 0,05 | 1,97 | 2,40 |
| Chromatography | Ddx | 4 | 100,00 | 0,00 | 0,00 | 0,05 | 100,00 | 100,00 |
| Diadinoxanthin purification | | | | | | | | |
| Efficiency | | | | | | | | |
| Extract | Dtx | 4 | 100,00 | 0,00 | 0,00 | 0,05 | 100,00 | 100,00 |
| Total partitioning | Dtx | 4 | 95,99 | 3,99 | 1,99 | 0,05 | 89,65 | 100,00 |
| Partitioning 1 | Dtx | 4 | 92,64 | 3,87 | 1,94 | 0,05 | 86,48 | 98,81 |
| Partitioning 2 | Dtx | 4 | 3,35 | 0,37 | 0,19 | 0,05 | 2,75 | 3,94 |
| Partitioning 3 | Dtx | 4 | 0,00 | 0,00 | 0,00 | 0,05 | 0,00 | 0,00 |
| Chromatography | Dtx | 4 | 73,22 | 1,51 | 0,76 | 0,05 | 70,81 | 75,62 |
| Enrichment | | | | | | | | |
| Extract | Dtx | 4 | 6,04 | 1,54 | 0,77 | 0,05 | 3,60 | 8,49 |
| Total partitioning | Dtx | 4 | 20,85 | 4,67 | 2,33 | 0,05 | 13,42 | 28,28 |
| Partitioning 1 | Dtx | 4 | 29,90 | 6,70 | 3,35 | 0,05 | 19,25 | 40,55 |
| Partitioning 2 | Dtx | 4 | 4,23 | 1,28 | 0,64 | 0,05 | 2,19 | 6,27 |
| Partitioning 3 | Dtx | 4 | 0,00 | 0,00 | 0,00 | 0,05 | 0,00 | 0,00 |
| Chromatography | Dtx | 4 | 100,00 | 0,00 | 0,00 | 0,05 | 100,00 | 100,00 |
